# Ultra-Fast Response ALTP Thermal Gas Flow Sensor Based on Si_3_N_4_/AlN Composite Insulating Structure

**DOI:** 10.3390/mi17050584

**Published:** 2026-05-09

**Authors:** Guo Chen, Xi Chen, Ziwen Jin, Hongbo Tian, Ruipeng Zhao, Bowan Tao

**Affiliations:** 1 National Key Laboratory of Electronic Thin Films and Integrated Devices, University of Electronic Science and Technology of China, Chengdu 611731, China; chenguyg@163.com (G.C.); ziwenjin0520@163.com (Z.J.); thbthb123@126.com (H.T.); ruipengzhao@uestc.edu.cn (R.Z.); taobw@uestc.edu.cn (B.T.); 2 Guizhou Aerospace Intelligent Agriculture Co., Ltd., Guiyang 550000, China

**Keywords:** ALTP thermal gas flow sensors, Si_3_N_4_/AlN composite films, response time, high-speed gas flow

## Abstract

Due to the intrinsic fast response characteristic of the atomic layer thermopile (ALTP) structure compared to the conventional thin film thermopile, the ALTP thermal gas flow sensor shows encouraging potential in fast gas flow detection. However, the thermal conduction delay between the ALTP film and the heating film still restricts the response speed. In this work, the prepared Si_3_N_4_/AlN composite films significantly reduced the thickness of the insulating layer while ensuring reliable insulation properties. Therefore, the response time of the ALTP thermal gas flow sensor was significantly reduced to 0.1 ms. Meanwhile, the sensor also demonstrated reliable and regular response signals in the tests of different gases, with different thermal sensitivity S*(N_2_) > S*(O_2_) > S*(Ar), conforming to the characteristics of thermal gas flow sensors. This work may promote the application of the ALTP sensor in high-speed gas flow detection.

## 1. Introduction

Gas flow measurement plays a significant role in industrial production, energy management, medical monitoring and other fields. Therefore, the gas flowmeters have been widely applied in various systems, such as target detection [1], respiratory monitoring during biomedical surgeries [2], drug delivery [3], liquid distribution systems [4], gas metering [5], and semiconductor manufacturing [6]. During the etching and deposition processes, the gas pulse valve must demonstrate a rapid response to fluctuations in gas flow to avoid defects on the wafer. Furthermore, the fuel flow of the engine must be matched with the instantaneous intake volume to optimize combustion efficiency.

Thermal gas flow sensors have the advantages of no moving parts, small size, low pressure loss and wide range, and have received extensive attention. Currently, the mainstream thermal gas flow sensors include hot-wire type [7], calorimetric type [8], and thermopile type [9]. The hot-wire flowmeter mainly reflects the change in gas flow through the current increment or change in resistance, but its long-term stability is relatively poor. Calorimetric flowmeters reflect the changes in gas flow by calculating the temperature difference between the upstream and downstream of the heat source, but there is a significant thermal delay phenomenon. The core of the thermopile flowmeter is to utilize the characteristic that thermoelectric materials generate potential differences under a temperature gradient, but the independent thermal resistance layer results in a bottleneck in its response time [10,11].

**Figure 1:**
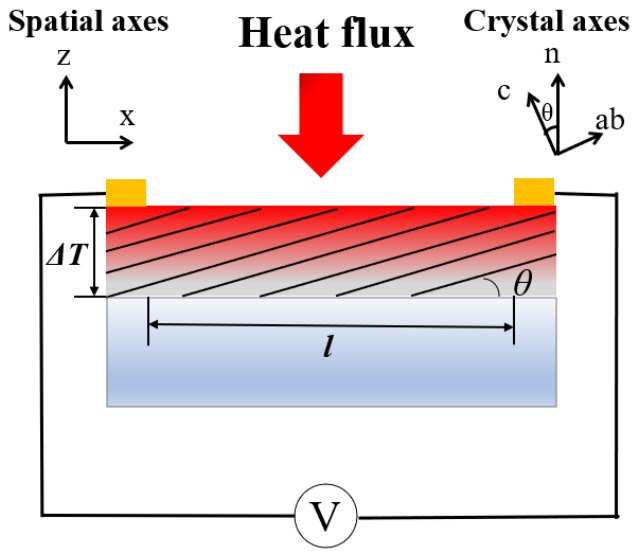
Schematic diagram of ALTP.

Compared with the traditional thermopile structure, the novel atomic layer thermopile (ALTP) has a significant advantage in response speed. As shown in Figure 1a, the ALTP structure is only a single inclined thin film with thermoelectric anisotropy. After the surface of the ALTP film absorbs heat flux, a temperature gradient is rapidly established in the direction of the film thickness. Based on the transverse thermoelectric effect, a transverse thermoelectric potential perpendicular to the temperature gradient is generated along the inclined direction of the film. Since the film thickness is usually hundreds of nanometers, and it serves as both a thermal resistance unit and a sensitive unit, ALTP has a significantly faster response speed than traditional thermopile structures. The transverse thermoelectric potential expression of ALTP is as follows [12]:

(1) $$U_{x}=l\cdot sin2a\left(S_{ab}-S_{c}\right)\Delta T_{z}/2d$$

where *a* is the inclined angle, *l* is the film length, *d* is the film thickness, Δ*T*_z_ is the temperature difference between the upper and lower surfaces of the film, and *S_ab_* and *S_c_* are the Seebeck coefficients in the direction of the *ab*-plane and along the *c*-axis, respectively.

Based on the fast response characteristics of ALTP, Tian et al. designed and fabricated the ALTP thermal gas flow sensor [10]. The device, from bottom to top, consists of an inclined single crystal substrate, ALTP film (La_1−x_Ca_x_MnO_3_, LCMO), MgO insulating layer, and heating layer (Pt film). When the gas flows through the Pt heat-generating film, the heat flux flowing from top to bottom through the LCMO film changes. Therefore, the change in its transverse thermoelectric potential can reflect the gas flow rate. The experimental results show that the response time of the ALTP thermal gas flow sensor reaches 0.32 ms, which is superior to other types of thermal gas flow sensors reported in the literature and has obvious advantages in rapid gas flow detection [11,13,14,15,16,17,18,19,20,21,22,23]. However, due to the relatively large thickness of the MgO insulating layer, the transmission of thermal signals is significantly delayed, so the current response speed still has a clear gap compared with that of sole ALTP films.

In this work, to improve the response speed of the ALTP thermal gas flow sensor, a composite Si_3_N_4_/AlN structure is adopted to reduce the thickness of the insulation layer, with the expectation of obtaining an improved response speed. The experimental results show that the 2 μm Si_3_N_4_/AlN film shows good insulation performance, so the sensor can respond regularly to the flow rate changes for different gases (N_2_, O_2_, Ar). Moreover, due to the different thermodynamic parameters in these gases, the thermal sensitivities of the sensor for N_2_, O_2_, and Ar are 0.181, 0.155, and 0.148 (mV/(L/min·W)), respectively, which is consistent with the characteristics of the thermal gas flow sensor. Due to the reduced thickness of the insulation layer, the response time of the thermal gas flow sensor is reduced from 0.32 ms to 0.1 ms. The above results further demonstrate the advantages of the ALTP thermal gas sensor in rapid flow detection and also show that optimizing the insulation layer structure is an effective means to improve its response speed.

## 2. Materials and Methods

**Figure 2:**
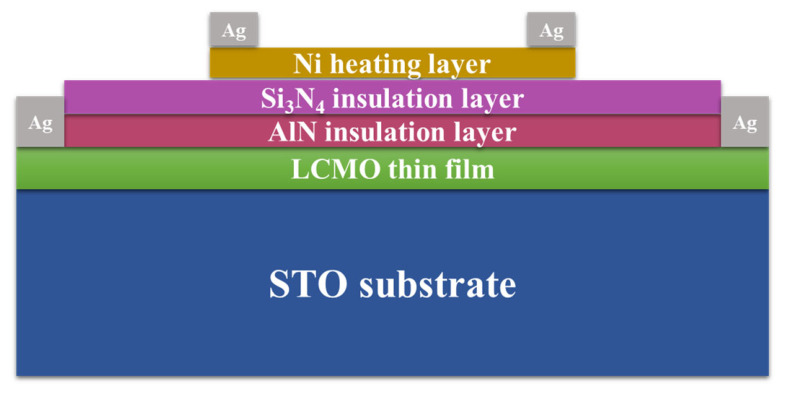
The structural schematic diagram of the ALTP thermal gas flow sensor.

Figure 2 shows a schematic diagram of the fabricated ALTP thermal gas flow sensor, including STO substrate, LCMO thin film, Si_3_N_4_/AlN insulation layer and Ni heating layer from bottom to top. LCMO thin film was fabricated on the miscut 12° SrTiO_3_ (STO) (001) single crystal through metal–organic chemical vapor deposition (MOCVD) at an optimized growth temperature of 830 °C. The precursor system was composed of La(DPM)_3_, Ca(DPM)_2_, and Mn(DPM)_3_ metal–organic compounds (Wuhan CVD Science & Technology Co., Ltd., Wuhan, China), which had been dissolved in tetrahydrofuran (THF) to form homogeneous precursor solutions. To avoid mutual interference between the thermoelectric signals of LCMO and the thermal excitation signals of the heating layer, AlN and Si_3_N_4_ films were grown, respectively, by mid-frequency reactive sputtering and radio frequency magnetron sputtering techniques. The process parameters are outlined in Table 1. Ni exhibits comparable resistivity and thermal conductivity with Pt, but it is less costly, so choosing nickel as the heating layer here. Ni thin film was prepared on the insulating layer by a high-vacuum evaporation coating system. The crystal structure and inclined orientation of the LCMO thin films were studied by X-ray diffraction (XRD). The surface morphologies of the LCMO thin film and the cross-section of the sensor were characterized by a field emission scanning electron microscope (FESEM). Elemental composition of the LCMO film and insulating layer was measured by energy dispersive X-ray spectroscopy (EDS).

## 3. Results

**Figure 3:**
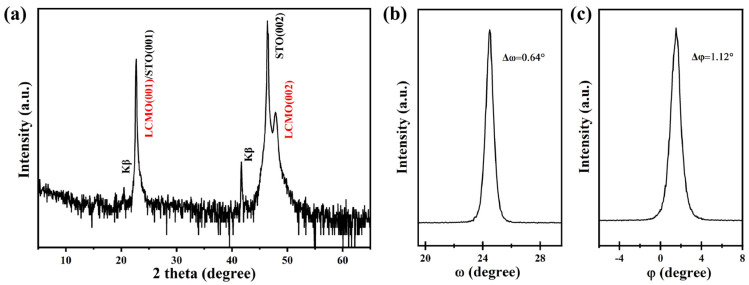
(**a**) The XRD 2*θ* pattern of LCMO thin film on STO substrate, (**b**) *ω*-scanning on the LCMO (002) peak, and (**c**) *φ*-scanning of the LCMO (101) peak.

Figure 3a shows the XRD 2*θ* pattern of LCMO thin film prepared on the miscut 12° STO (001) substrate using an offset model. It can be clearly seen that only LCMO (00L) diffraction peaks are observed except for (00L) diffraction peaks of the STO substrate, indicating a highly c-axis-oriented LCMO thin film. The *ω*-scanning and *φ*-scanning curves for LCMO thin film are shown in Figure 3b and Figure 3c, respectively, to characterize the textures of inclined epitaxial LCMO thin film on out-of-plane and in-plane. The full width at half maximum (FWHM, Δ*ω* and Δ*φ*) values determined by the *ω*-scanning and *φ*-scanning curves are 0.64° and 1.12°, respectively, showing that the prepared LCMO thin film has good crystallization quality and biaxial textures.

**Figure 4:**
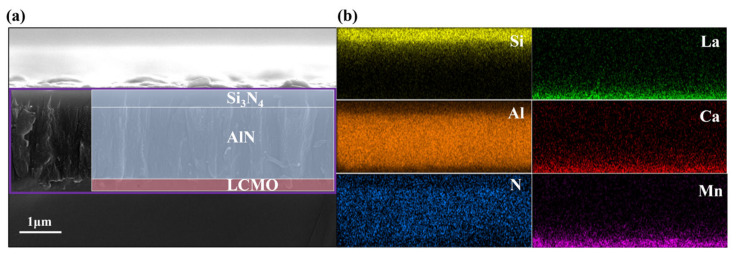
(**a**) The cross-sectional SEM image and (**b**) EDS mapping image of Si_3_N_4_/AlN/LCMO film.

**Figure 5:**
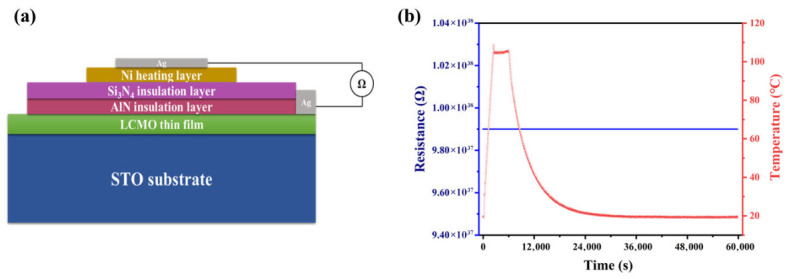
(**a**) The schematic diagram and (**b**) the result of insulation performance measurement.

Figure 4a,b show the cross-sectional morphology and EDS mapping image of Si_3_N_4_/AlN/LCMO, respectively, with a clear interface for each layer of film, and the thickness of the LCMO film and the Si_3_N_4_/AlN film is approximately 300 nm and 2 μm, respectively. The insulating properties of the insulating layer are pivotal in determining the effectiveness of the barrier between the thermal excitation signal of the heating layer and the thermoelectric signal of the sensitive layer. This barrier is critical to the normal operation of the sensor, so the insulating properties of the insulating layer were characterized. Figure 5a shows the schematic diagram of the insulation performance test for the insulation layer; the two electrodes are located in the LCMO and Ni heating layers, respectively. The experimental results indicate the insulation properties of the insulating layer remained stable with no substantial fluctuations (Figure 5b), meeting the requirements of the ALTP thermal gas flow sensor.

**Figure 6:**
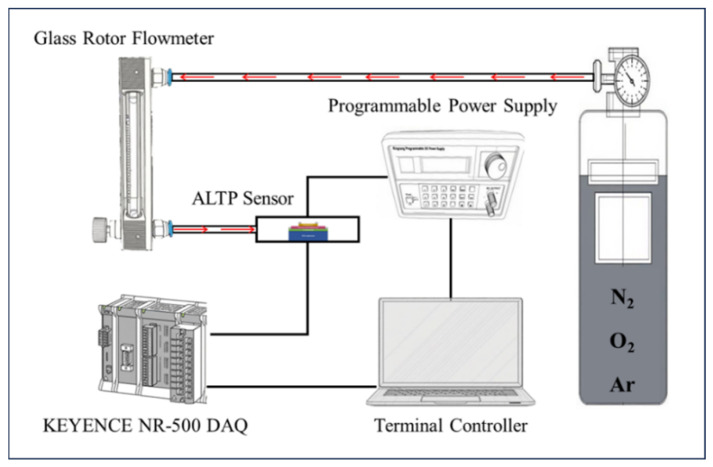
The illustration diagram of the test system.

The ALTP thermal flow sensor was evaluated using a self-built gas flow testing platform, as shown in Figure 6. Three gases, namely N_2_, O_2_ and Ar, were selected to test the performance of the sensor. A constant heat flux flowing from top to bottom through the LCMO film is generated by applying a fixed power to the Ni film, while a constant transverse thermoelectric potential is produced at both ends of the LCMO film in the meantime. When the gas flow passes over the surface of the sensor, it will carry away the heat of the Ni heating layer; that is, the heat flux flowing through the LCMO film changes, and the corresponding lateral thermoelectric potential also changes. Therefore, the voltage variation in the LCMO film can reflect the gas flow rate.

**Figure 7:**
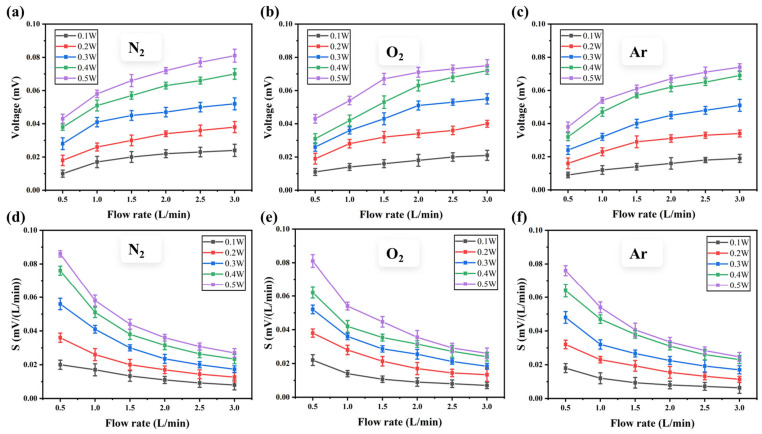
Heating power range 0.1 W to 0.5 W and gas flow rate of N_2_, O_2_, and Ar from 0.5 L/min to 3.0 L/min. (**a**–**c**) the response curve of the ALTP sensors, (**d**–**f**) the sensitivity curve.

To calibrate the ALTP thermal gas sensor, we applied five different heating powers to the heating layer through a programmable power supply, ranging from 0.1 W to 0.5 W. The gas flow rate passing through the sensor surface is adjusted by a flowmeter from 0.5 L/min to 3 L/min. Figure 7a–c represent the response curves of the sensor for N_2_, O_2_ and Ar at different powers and gas flow rates, respectively. The results show that, at the same power, as the gas flow rate increases, the response voltage of the ALTP thermal gas sensor also increases. However, the growth rate of the response voltage gradually decreases rather than in a linear relationship. According to the principle of thermal diffusion [24]:

(2) $$h\propto \sqrt{v}$$

where *h* is the convective heat transfer coefficient, and *v* is the gas flow rate. The heat carried away by the gas flow (heat loss) shows a nonlinear positive correlation with the gas flow rate, supporting the above measurement results. Additionally, under the same gas flow rate, the greater the heating power, the greater the change in temperature gradient of the LCMO film, and the greater the response voltage signal. As shown in Figure 7d–f, the sensitivity *S* of the sensor is highest when the flow rate is 0.5 L/min, with 0.086 mV/(L/min), 0.081 mV/(L/min), 0.076 mV/(L/min) for N_2_, O_2_ and Ar, respectively. Then, it shows a downward trend with increasing flow rate.

**Figure 8:**
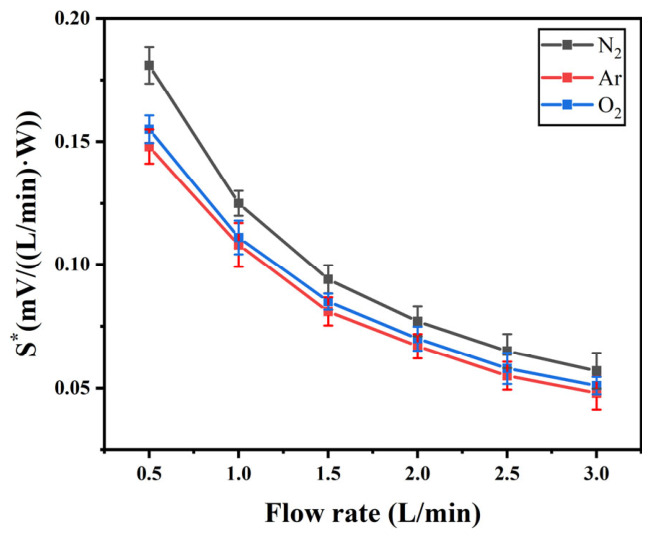
The thermal sensitivity curves of N_2_, O_2_, and Ar.

Figure 8a shows the changes in the thermal sensitivity S* of the sensor for three gases at different flow rates, where S*(N_2_) > S*(O_2_) > S*(Ar) within the flow rate range from 0.5 L/min to 3 L/min. The thermodynamic parameters of N_2_, O_2_ and Ar are shown in Table 2. *C*_p_ is the constant-pressure heat capacity, *μ* is the dynamic viscosity, *λ* is the thermal conductivity, and *ρ* is the density of the gas. According to the heat transfer formula, the cooling effect of gas on a thermal flow sensor can be simplified as [25]:

(3) $$Q\propto \lambda \cdot \Delta T+\rho \cdot C_{p}\cdot v\cdot \Delta T$$

Among them, *C_p_* and *λ* play a major role, and *ρ* and *v* are secondary factors. Therefore, there is the best and worst heat transfer efficiency for N_2_ and Ar, respectively, which is consistent with the experimental results.

**Figure 9:**
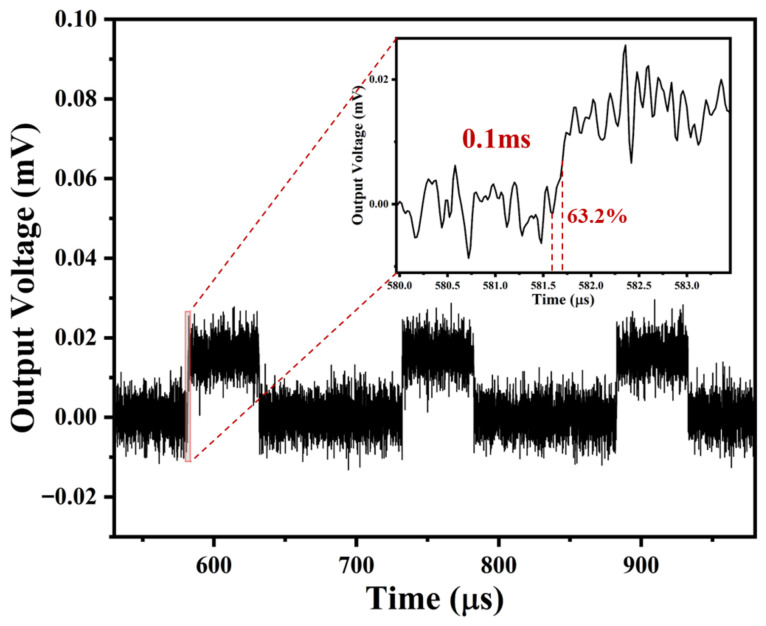
The dynamical response to the ALTP thermal gas sensor.

Response time is an important parameter for evaluating the performance of sensors. The response time of the ALTP thermal gas sensor is measured by applying a square wave current to the heating layer, which is defined as the time from the initial value to the stable value of response voltage. As shown in Figure 9, according to the definition of the response time of the flow system described in ref. [20], the response time of approximately 0.1 ms can be estimated, significantly faster than that in ref. [10]. The main reasons for this can be summarized as follows: (1) The thermal conductivity of AlN and Si_3_N_4_ is stronger than that of MgO; (2) The thickness of the insulating layer has been reduced. Figure S1 presents the simulation results of the transient response for the ALTP thermal gas flow sensor with different thicknesses of AlN insulating layers, indicating that reducing the thickness of the insulating layer significantly helps to enhance the response speed. At the same time, obvious noise signals were observed, so reducing signal noise is also a problem that needs to be given special attention [26].

## 4. Conclusions

In conclusion, we fabricated an improved ALTP thermal gas flow sensor based on the transverse thermoelectric effect of inclined LCMO thin film. The signal responses of three gases, N_2_, O_2_, and Ar, at heating powers ranging from 0.1 W to 0.5 W and gas flow rates ranging from 0.5 L/min to 3.0 L/min were investigated. Due to different heat transfer efficiency in three gases, the sensor shows different thermal sensitivity, S*(N_2_) > S*(O_2_) > S*(Ar). By improving the insulation layer structure (Si_3_N_4_/AlN film) to reduce the thickness, the response speed of the sensor has been further enhanced, with approximately 0.1 ms. The above results further show the potential of ALTP in fast gas flow detection.

## Figures and Tables

**Table 1:** The process parameters of AlN and Si_3_N_4_ films.

Film	Thickness (μm)	Sputtering Power (W)	Deposition Pressure (Pa)	Substrate Temperature (°C)	Deposition Rate (μm/h)	Gas Ratios (sccm)
AlN	~1.6	2000	0.4	350	1.2	N_2_: 100
Si_3_N_4_	~0.4	150	0.4	RT	0.25	Ar/N_2_: 30/7.5

**Table 2:** The thermodynamic parameters of N_2_, O_2_, and Ar.

Gas	*C_p_* (J/(kg·K))	*μ* (mPa·s)	*λ* (W/(m·K))	*ρ* (kg/m^3^)
N_2_	1041	0.0175	0.026	1.145
O_2_	920	0.0204	0.026	1.308
Ar	519	0.0227	0.0177	1.663

## Data Availability

The original contributions presented in the study are included in the article and Supplementary Materials, further inquiries can be directed to the corresponding author.

## Supplementary Materials

The following supporting information can be downloaded at: https://www.mdpi.com/article/10.3390/mi17050584/s1, Figure S1. The simulation results of the transient response for the ALTP thermal gas flow sensor with different thicknesses of AlN insulating layers. (a) transient response curve, (b) transient response time.
