# Supplementary material for: Ultra-Fast Response ALTP Thermal Gas Flow Sensor Based on Si3N4/AlN Composite Insulating Structure
Source: Micromachines (Basel). 2026 May 9;17(5):584. doi: 10.3390/mi17050584 (PMC13209437; doi:10.3390/mi17050584)
Supplement: Supplementary file 1 [file micromachines-17-00584-s001.zip › micromachines-4282737-supplementary.pdf]

micromachines

Supplementary Materials

Ultra-Fast Response ALTP Thermal Gas Flow Sensor Based on Si_3N_4/AlN Composite Insulating Structure

Guo Chen [1,2], Xi Chen [1,*], Ziwen Jin [1], Hongbo Tian [1], Ruipeng Zhao [1] and Bowan Tao [1]

[1] National Key Laboratory of Electronic Thin Films and Integrated Devices, University of Electronic Science and Technology of China, Chengdu 611731, China; chenguyg@163.com (G.C.); ziwenjin0520@163.com (Z.J.); thbthb123@126.com (H.T.); ruipengzhao@uestc.edu.cn (R.Z.); taobw@uestc.edu.cn (B.T.)

[2] Guizhou Aerospace Intelligent Agriculture Co., Ltd., Guiyang 550000, China

* Correspondence: chenxi2024@uestc.edu.cn

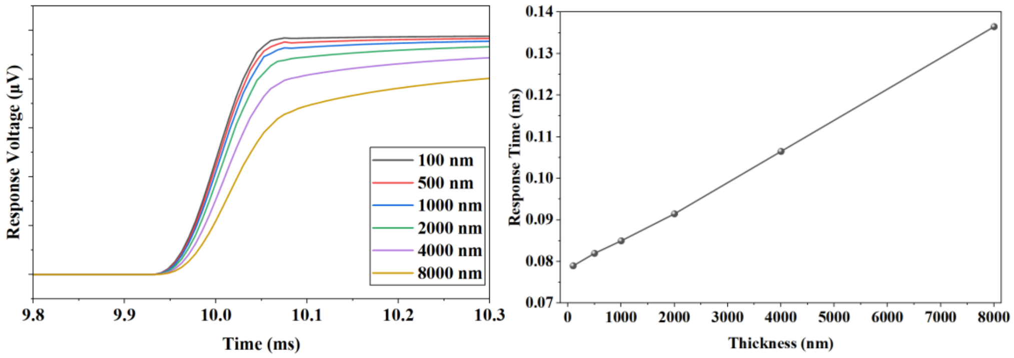

(**a**) (**b**)

Figure S1. The simulation results of the transient response for the ALTP thermal gas flow sensor with different thicknesses of AlN insulating layers. (**a**) transient response curve, (**b**) transient response time.
